# M-SAC-VLADNet: A Multi-Path Deep Feature Coding Model for Visual Classification

**DOI:** 10.3390/e20050341

**Published:** 2018-05-04

**Authors:** Boheng Chen, Jie Li, Gang Wei, Biyun Ma

**Affiliations:** The National Engineering Technology Research Center for Mobile Ultrasonic Detection, School of Electronics and Information Engineering, South China University of Technology, Guangzhou 510641, China

**Keywords:** deep convolutional network, deep feature coding network, multi-path feature coding network, sparsely-adaptive and covariance VLAD coding, visual classification

## Abstract

Vector of locally aggregated descriptor (VLAD) coding has become an efficient feature coding model for retrieval and classification. In some recent works, the VLAD coding method is extended to a deep feature coding model which is called NetVLAD. NetVLAD improves significantly over the original VLAD method. Although the NetVLAD model has shown its potential for retrieval and classification, the discriminative ability is not fully researched. In this paper, we propose a new end-to-end feature coding network which is more discriminative than the NetVLAD model. First, we propose a sparsely-adaptive and covariance VLAD model. Next, we derive the back propagation models of all the proposed layers and extend the proposed feature coding model to an end-to-end neural network. Finally, we construct a multi-path feature coding network which aggregates multiple newly-designed feature coding networks for visual classification. Some experimental results show that our feature coding network is very effective for visual classification.

## 1. Introduction

Deep learning models have gained great attention in the field of computer vision, including visual classification [1,2,3,4,5,6,7,8], super resolution [9,10], semantic segmentation [11,12], object detection [13,14,15] and visual tracking [16]. Compared with the traditional statistical learning methods, deep learning models have two main advantages: (1) based on end-to-end training manner, the networks parameters which are more suitable for the final task can be obtained; and (2) the deep network representation can provide a better description. The deep feature methods can significantly improve the performances over the conventional feature methods, such as scale invariant feature transform (SIFT) [17] feature method and histograms of gradients (HOG) [18] feature method.

Since the end-to-end training model and deep structure representation have great advantages, some recent papers embed the domain knowledge of conventional statistical learning models into the deep neural network and train the entire model by an end-to-end manner. The new neural networks not only inherit the domain expertise but also make all the parameters more suitable for the final application tasks. Representative works include the following. Zuo et al. [19] proposed a novel iteration-wise $l_{p}$-norm regularizer which is from the maximum a posterior (MAP) model to get the outstanding blind de-convolution results. Peng et al. [20] proposed a novel deep subspace clustering method with sparse prior to obtain the state-of-the-art clustering results. Wang et al. [21] proposed a novel end-to-end $l_{\infty }$ norm encoder to get the state-of-the-art hash results. Zheng et al. [12] treated the conditional random field as the recursive neural network (RNN), and then plugged this new structure RNN into a deep convolutional neural network (CNN) to obtain the state-of-the-art image segmentation performances. Wang et al. [22] extended the traditional dictionary pairs classifier [23] to an end-to-end classifier layer, and then embedded this new layer into a deep CNN to get the state-of-the-art object detection results. In [10], the domain expertise of sparse coding has been combined with the merits of the deep neural network to improve the super-resolution performance. The computation steps and the optimization procedures of some statistical learning methods can be considered as new structure layers which provide some interpretations for deep learning models.

Feature coding [24,25] is a popular statistical learning method for visual classification. In the traditional feature coding framework, feature coding is an important step, which connects feature extraction and feature pooling. Feature coding also greatly affects the image recognition result. Many effective feature coding methods have been proposed. The representative feature coding models include sparse coding [26] model, convolutional sparse coding [27,28,29] model, locality constrained coding (LLC) [30] model, soft coding [31] model, hard coding [32] model, salient coding [33] model, Fisher Vector (FV) coding [34] model and Vector of Locally Aggregated Descriptor (VLAD) coding [35] model. Since all the algorithmic components (feature extraction, dictionary learning, feature coding and classifier training) in the conventional feature coding approaches are independent, the learned parameters may be suboptimal for visual classification. Besides, the SIFT [17] features in conventional feature coding methods are not good descriptors. Recently, the conventional VLAD coding model is extended to a deep network which is called NetVLAD [36]. The NetVLAD layer is jointly trained with a CNN to obtain the excellent retrieval and classification results. Besides, the NetVLAD model has demonstrated its effectiveness in the field of action classification [37].

Although the NetVLAD model has been proposed, the NetVLAD method only aggregates the first order statistical information from the spatial scale, thus the discriminative power of the NetVLAD model is not fully researched. In this paper, a discriminative sparsely-adaptive and covariance VLAD (SAC-VLAD) approach is proposed. Since each trainable parameter in SAC-VLAD coding is differentiable for the final classification loss, we derive the back propagation models of each trainable parameter and design a new deep feature coding network called SAC-VLADNet. By using the back-propagation algorithm to minimize the final classification loss, the trained SAC-VLADNet can be more suitable for image classification task. Moreover, we construct a multi-path SAC-VLADNet (M-SAC-VLADNet) which aggregates multiple newly-designed SAC-VLADNets to further improve the visual classification performance. Since our networks can effectively integrate the domain expertise of the new discriminative feature coding and the deep neural network, the newly-designed SAC-VLADNet and M-SAC-VLADNet can introduce more interpretations and discriminations into the deep learning models.

The contributions of the proposed model are summarized in the following three aspects.

The first contribution is the newly-designed sparsely adaptive and covariance VLAD network. The weight coefficient of the original NetVLAD [36] method is the soft assignment coding. In our SAC-VLADNet, a new coding method called sparsely adaptive soft assignment coding (SASAC) is used as the weight coefficient. The SASAC layer can be considered as a variant of multidimensional Gaussian probability density function and adaptively learn all the parameters (dictionary and variance) by an end-to-end fashion. Besides, many works show that the sparse features are helpful for improving the image classification performance. To obtain the sparse weight coefficient, the SASAC layer only holds the largest *T* probabilities and enforces other small probabilities to be zeros. To the best of our knowledge, the end-to-end SASAC layer is not studied in current deep neural networks. We design a new end-to-end layer by a new coding method. The original NetVLAD method used the first-order VLAD coding to obtain the final representation. Our network uses the covariance VLAD coding to obtain the interactive feature representation. The final feature representation of the proposed network is the concatenation of the first-order and the covariance feature coding. Besides, the proposed network extends the affine subspace method in [38] to a 1×1 convolutional layer which reduces the dimension of the coding.

The second contribution is the proposed Multi-path SAC-VLADNet. The existing feature coding networks only extract the features of the last convolutional layer of a deep convolutional network to compute the feature codings, thus these models can not take full advantage of the convolutional representations for visual classification. To take full advantage of multiple levels representations, the proposed M-SAC-VLADNet uses a novel manner to aggregate multiple SAC-VLAD layers. In the M-SAC-VLADNet, we first extract the convolutional features from multiple layers. Next, we obtain the corresponding SAC-VLAD coding in each convolutional feature. Finally, we aggregate all the SAC-VLAD codings to construct the final multi-path feature coding network which is also an end-to-end feature coding model. The M-SAC-VLADNet can simultaneously use the low, middle and high level features to train multiple feature coding networks, thus will be more discriminative than the single level feature coding network.

The third contribution is that the back propagation function of each new layer is derived. Based on the back propagation algorithm, all the learnable parameters can be obtained. The back propagation models of affine subspace layer and covariance VLAD layer are easily obtained. The SASAC layer is a completely new structure layer, thus we will detailedly discuss the back propagation model of the SASAC layer. Various visual classification experiments will show the superiorities of the new layers. In addition, some visual recognition results demonstrate that SAC-VLADNet is evidently better than SAC-VLAD, and M-SAC-VLADNet is better than SAC-VLADNet. These results demonstrate the superiorities of the end-to-end model and the proposed multi-path feature coding network. We also give some detailed experimental results of our network and other state-of-the-art models to show the advantages of our network.

The remainder of this paper is organized as follows. Section 2 briefly introduces the traditional feature coding framework, the CNN feature for feature coding network and the end-to-end NetVLAD model. Section 3 presents the SAC-VLADNet and the M-SAC-VLADNet. Section 4 gives the experimental comparisons between the proposed model and other state-of-the-art models. Finally, Section 5 concludes this paper.

## 2. Related Work

In this section, the introduction of the traditional feature coding framework for visual classification is first given. Next, the introduction of the CNN feature in the feature coding network is given. Finally, the introduction of the NetVLAD method is given.

### 2.1. The Conventional Feature Coding Framework for Image Recognition

**Figure 1:**
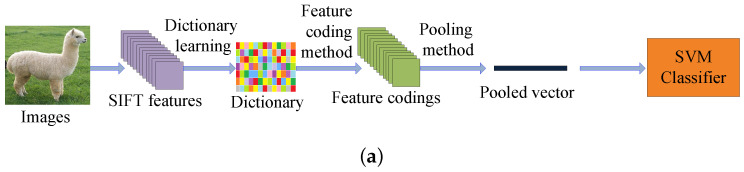

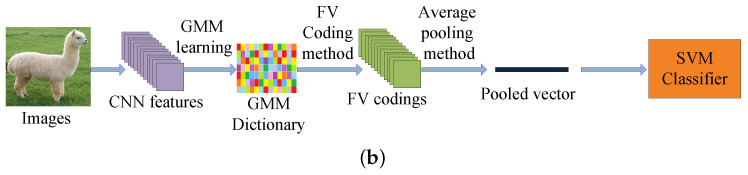
(**a**) The traditional feature coding framework; (**b**) The block diagram of the FV-CNN [39].

The traditional feature coding framework can be divided into five steps: (1) extracting the SIFT [17] features from all the images; (2) solving an minimization problem from all the training SIFT representations to obtain a dictionary; (3) computing the feature codings by a specific feature coding method; (4) pooling the feature codings to get the pooled vectors; and (5) training the final support vector machine (SVM) classifier by the pooled vectors to get the classification result. The block diagram of the traditional feature coding framework for image recognition is shown in Figure 1a.

### 2.2. The CNN Feature for Feature Coding Network

Since the SIFT [17] feature does not have the strong image representation ability, the image classification performances of the traditional feature coding methods are not always satisfactory. Recently, some feature coding models which utilize the CNN features are proposed. Compared with the shallow SIFT feature, the CNN feature is a deeper and more descriptive representation of the original image. In visual classification, the CNN based feature coding networks are obviously better than the SIFT based feature coding methods. Fisher Vector with CNN (FV-CNN [39]) is a representative feature coding network which is based on the CNN feature. FV-CNN [39] trains a gaussian mixture model (GMM) dictionary by the CNN feature and obtains the Fisher Vector (FV) codings by the trained GMM dictionary. The block diagram of the FV-CNN [39] for visual classification is shown in Figure 1b.

To obtain the CNN features of a feature coding network, all the images need to pass through a CNN which is pre-trained on the large scale ImageNet [40] dataset. The most useful features extracted from the pre-trained CNN are the feature of the last convolutional layer and the feature of the last fully connected layer [39]. In the proposed model, we extract the feature of a specific convolutional layer to train our feature coding network.

For a size-*s* RGB image $I\in R^{S\times S\times 3}$, the extracted feature of a specific convolutional layer of a deep CNN can be expressed as $F\in R^{O\times O\times D}$, and *D* represents the number of the convolutional kernels of a specific convolutional layer. *O* represents the size of the convolutional feature. *F* can also be viewed as a feature set which contains M=O×O convolutional descriptors, and each descriptor is *D*-dimensional.

### 2.3. The End-to-End NetVLAD Model

The NetVLAD model uses the last convolutional feature to train the NetVLAD layer, thus the descriptor set $F_{i}={\left\{f_{ij}\right\}}_{j=1}^{M}$ represents the last convolutional feature of the *i*th image $I_{i}$, and the total number of the images is *N*. $f_{ij}\in R^{D\times 1}$ is the *j*th descriptor of $F_{i}$. Besides, the NetVLAD model uses *K* visual words ${\left\{c_{k}\right\}}_{k=1}^{K}$ ($c_{k}\in R^{D\times 1}$) as the dictionary.

For $F_{i}$, the final VLAD vector is K×D-dimensional and can be expressed as:

(1) $$\Psi \left(F_{i}\right)=\sum_{j=1}^{M}\Psi (f_{ij})$$

where $\Psi \left(f_{ij}\right)\in R^{KD\times 1}$ is the VLAD representation of $f_{ij}$. The expression of $\Psi (f_{ij})$ is:

(2) $$\Psi \left(f_{ij}\right)={\left[\varphi {\left(f_{1}^{ij}\right)}^{T},\cdots ,\varphi {\left(f_{k}^{ij}\right)}^{T},\cdots ,\varphi {\left(f_{K}^{ij}\right)}^{T}\right]}^{T}$$

where the sub vector $\varphi \left(f_{k}^{ij}\right)\in R^{D\times 1}$ in Equation (2) is written as:

(3) $$\varphi \left(f_{k}^{ij}\right)=\lambda_{ij}\left(k\right)\left(f_{ij}-c_{k}\right)$$

where $\lambda_{ij}\left(k\right)$ represents the weight coefficient of $c_{k}$ and $f_{ij}$. In the traditional VLAD [35] model, hard assignment coding is used as the weight coefficient. In the NetVLAD model, soft assignment coding [31] is used as the weight coefficient, and the soft assignment coding is written as:

(4) $$\lambda_{ij}\left(k\right)=\frac{e^{-\frac{\left|\right|f_{ij}-c_{k}{\left|\right|}_{2}^{2}}{2\sigma^{2}}}}{\sum_{k^{\prime }=1}^{K}e^{-\frac{\left|\right|f_{ij}-v_{k^{\prime }}{\left|\right|}_{2}^{2}}{2\sigma^{2}}}}$$

where $\left||.\right|{|}_{2}$ is the $l_{2}$ norm of a vector. $\sigma^{2}$ represents the covariance coefficient which controls the decay of the response with the magnitude of the distance. As Equation (4) shows, the soft assignment coding is a normalized weight coefficient which uses the sum of *K* probabilities as the denominator. After some simple transformations, Equation (4) can be decomposed into a 1×1 convolutional layer and a soft-max activation function layer. Based on Equations (1)–(4), the final expression of the NetVLAD model can be written as:

(5) $$V\left(F_{i}\right)\left(k,d\right)=\sum_{j=1}^{M}\frac{e^{w_{k}^{T}f_{ij}+\delta_{k}}}{\sum_{k^{\prime }=1}^{K}e^{w_{k^{\prime }}^{T}f_{ij}+\delta_{k^{\prime }}}}\left(f_{ij}\left(d\right)-c_{k}\left(d\right)\right)$$

where $\Psi \left(F_{i}\right)\left(k,d\right)$ represents the ${\left(\left(k-1\right)D+d\right)}^{th}$ element of $\Psi (F_{i})$(k=1,2,⋯,K;d=1,2,⋯,D). $f_{ij}\left(d\right)$ and $c_{k}\left(d\right)$ represent the $d^{th}$ (d=1,2,⋯,D) element of $f_{ij}$ and $c_{k}$ respectively.

**Figure 2:**
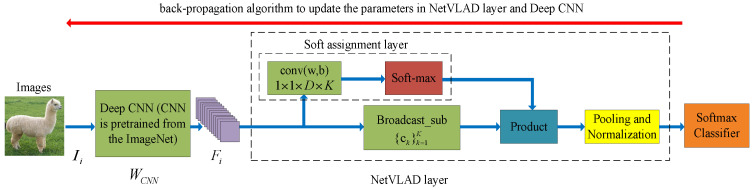
The network structure of the NetVLAD for visual classification. The blue arrow represents the feed-forward operation of the NetVLAD, and the red arrow represents the back-propagation operation of the NetVLAD.

The NetVLAD layer also uses the widely-used L2-normalization method and intra-normalization [41] method to obtain the final coding representation. The complete NetVLAD model for visual classification is illustrated in Figure 2.

## 3. The Proposed SAC-VLADNet

**Figure 3:**
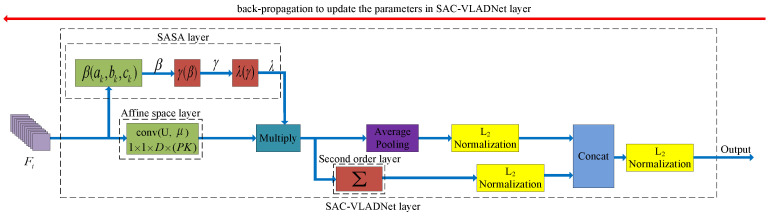
The network structure of the SAC-VLADNet layer. $F_{i}$ is the feature of the $i^{th}$ image in a specific convolutional layer. The blue arrow represents the feed-forward operation of the SAC-VLADNet layer, and the red arrow represents the back-propagation operation of the SAC-VLADNet layer. $\beta (a_{k},b_{k},v_{k})$, γ(β) and λ(γ) are Equations (A1)–(A3), respectively. Σ layer is the covariance statistic layer in Equation (14). conv(U,μ) is the 1×1 convolutional layer with the weight $\left\{U_{k}\right\}$ and the bias $\left\{\mu_{k}\right\}$. $a_{k}$, $b_{k}$, $v_{k}$, $U_{k}$ and $\mu_{k}$ (k=1,2,⋯,K) are the trainable parameters, which are obtained by the back propagation algorithm.

**Figure 4:**
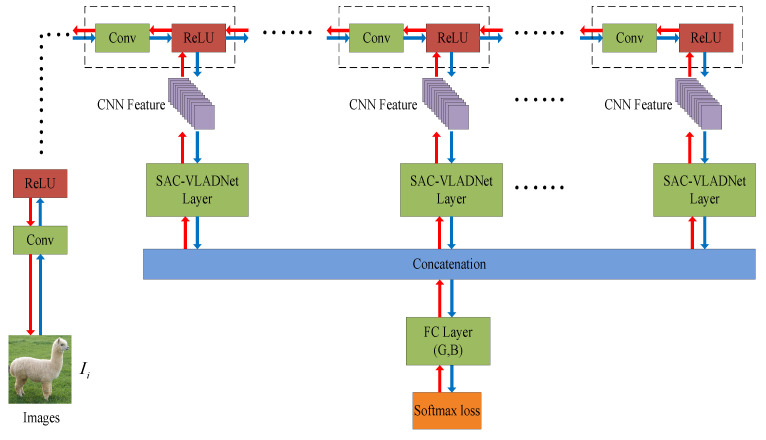
The network structure of the M-SAC-VLADNet. The blue arrow represents the feed-forward operation of the M-SAC-VLADNet, and the red arrow represents the back-propagation operation of the M-SAC-VLADNet. *G* and *B* are the weight and bias of the final softmax classifier.

In this section, the mathematical details of the SASAC layer, the affine subspace layer and the covariance layer in our SAC-VLADNet will be presented. We further propose the multi-path M-SAC-VLADNet which aggregates multiple SAC-VLADNet layers. The proposed SAC-VLADNet layer is shown in Figure 3. The proposed M-SAC-VLADNet for image classification is illustrated in Figure 4.

### 3.1. The Sparsely-Adaptive Soft Assignment Coding (SASAC) Layer

The NetVLAD model uses the soft assignment coding in Equation (4) as the weight coefficient. Equation (4) can be considered as a normalized probability. For each *i*, *j* and *k*, the probability of $f_{ij}$ and $c_{k}$ is $p_{ij}\left(k\right)=e^{-\frac{\left|\right|f_{ij}-c_{k}{\left|\right|}_{2}^{2}}{2\sigma^{2}}}$.

In the proposed network, we use the newly designed SASAC layer as the weight coefficient. The SASAC layer uses a multidimensional Gaussian probability density function (MGPDF) to define the probability of $f_{ij}$ and $c_{k}$. The MGPDF with Euclidean distance is written as:

(6) $$\left\{\begin{array}{c} \sigma_{k}=\sigma_{k1}\sigma_{k2}\cdots \sigma_{kD}\in R\\ \phi_{k}={\left[\sigma_{k1},\sigma_{k2},\cdots ,\sigma_{kD}\right]}^{T}\in R^{D\times 1}\\ p_{ij}\left(k\right)=\frac{1}{{\left(2\pi \right)}^{D/2}\sigma_{k}}e^{-\frac{1}{2}\left|\right|\left(f_{ij}-c_{k}\right)./\phi_{k}{\left|\right|}_{2}^{2}} \end{array}\right.$$

where ./ is the element wise division operation of two vectors, $\sigma_{k1},\sigma_{k2},\cdots ,\sigma_{kD}$ are the covariance parameters of $c_{k}$. Different from the standard MGPDF that directly computes $\sigma_{k}=\sigma_{k1}\sigma_{k2}\cdots \sigma_{kD}$, our SASAC layer uses a trainable parameter to replace $\sigma_{k}$. The trainable probability density function in SASAC layer is written as:

(7) $$p_{ij}\left(k\right)=\frac{1}{{\left(2\pi \right)}^{D/2}}e^{-\left|\right|a_{k}.*f_{ij}+b_{k}{\left|\right|}_{2}^{2}+v_{k}}$$

where .∗ is the element wise multiplication operation of two vectors, and $a_{k}\in R^{D\times 1}$, $b_{k}\in R^{D\times 1}$ and $v_{k}\in R$ are the trainable parameters. If we set $a_{k}$, $b_{k}$ and $v_{k}$ as the following parameters, Equation (7) will be exactly equivalent to Equation (6).

(8) $$\left\{\begin{array}{c} a_{k}=1./\left(\sqrt{2}\phi_{k}\right)\\ b_{k}=-c_{k}./\left(\sqrt{2}\phi_{k}\right)\\ v_{k}=\ln\left(\sigma_{k}\right) \end{array}\right.$$

However, in the SASAC layer, $a_{k}$, $b_{k}$ and $v_{k}$ are achieved by an end-to-end learning manner, instead of being directly constructed from the pre-computed expression in Equation (8).

Similar to the soft assignment coding in Equation (4), the SASAC layer also uses normalized probability to construct the weight coefficient. The normalized expression of Equation (7) is written as:

(9) $$\lambda_{ij}\left(k\right)=\frac{e^{-\left|\right|a_{k}.*f_{ij}+b_{k}{\left|\right|}_{2}^{2}+v_{k}}}{\sum_{k^{\prime }=1}^{K}e^{-\left|\right|a_{k^{\prime }}.*f_{ij}+b_{k^{\prime }}{\left|\right|}_{2}^{2}+v_{k^{\prime }}}}$$

For a certain *k*, if the probability $p_{ij}\left(k\right)$ is very small, this unreliable probability will affect the classification performance of the model. Besides, many works show that the sparse codings are helpful for improving the image classification performance. To eliminate the adverse impacts of the unreliable probabilities and obtain the sparse weight coefficient, the SASAC layer only considers the largest *T* probabilities and forces other small probabilities to be 0. The final expression of our SASAC layer is the following expression.

(10) $$\lambda_{ij}\left(k\right)=\left\{\begin{array}{c} \frac{e^{-\left|\right|a_{k}.*f_{ij}+b_{k}{\left|\right|}_{2}^{2}+v_{k}}}{\sum_{k^{\prime }\in S_{T}\left(f_{ij}\right)}e^{-\left|\right|a_{k^{\prime }}.*f_{ij}+b_{k^{\prime }}{\left|\right|}_{2}^{2}+v_{k^{\prime }}}},\;k^{\prime }\in S_{T}\left(f_{ij}\right)\\ 0,\;otherwise \end{array}\right.$$

where $S_{T}\left(f_{ij}\right)$ is a set that satisfies the following conditions:

(11) $$\left\{\begin{array}{c} S_{T}\left(f_{ij}\right)\cup \bar{S_{T}\left(f_{ij}\right)}=\left\{1,2,\ldots ,K\right\}\\ Card(S_{T}\left(f_{ij}\right))=T\leq K\\ \forall k\in S_{T}\left(f_{ij}\right),\forall k^{\prime }\in \bar{S_{T}\left(f_{ij}\right)},\\ e^{-\left|\right|a_{k}.*f_{ij}+b_{k}{\left|\right|}_{2}^{2}+v_{k}}\geq e^{-\left|\right|a_{k^{\prime }}.*f_{ij}+b_{k^{\prime }}{\left|\right|}_{2}^{2}+v_{k^{\prime }}} \end{array}\right.$$

where $\bar{S_{T}\left(f_{ij}\right)}$ is the complementary set of $S_{T}\left(f_{ij}\right)$ in the set {1,2,⋯,K}. $Card(S_{T}\left(f_{ij}\right))$ is the number of elements in $S_{T}\left(f_{ij}\right)$.

It is easy to see that the soft assignment coding in Equation (4) can be considered as a special case of Equation (10) when $a_{k}={\left[\frac{1}{\sqrt{2}\sigma },\frac{1}{\sqrt{2}\sigma },\cdots ,\frac{1}{\sqrt{2}\sigma }\right]}^{T}\in R^{D\times 1}$, $b_{k}=-\frac{1}{\sqrt{2}\sigma }c_{k}\in R^{D\times 1}$, $v_{1}=v_{2}=\cdots =v_{K}$ and T=K. Our SASAC layer in Equation (10) can adaptively learn all the parameters ($a_{k}$, $b_{k}$ and $v_{k}$) based on a normalized MGPDF and obtain more sparse weight coefficient than the soft assignment coding layer in Equation (4). The SASAC layer is differentiable, thus the SASAC layer can be trained in an end-to-end method which can obtain the more suitable parameters for image classification. The SASAC layer is a new neural network layer which incorporates the domain knowledge of the sparse MGPDF and the deep learning model. To the best of our knowledge, the end-to-end SASAC layer is not studied in the previous deep neural network. In this paper, we first embed the end-to-end SASAC layer into a deep neural network for image classification.

### 3.2. The End-to-End Affine Subspace Layer

The original NetVLAD model exploits the PCA algorithm for dimension reduction. The proposed network exploits the affine subspace method in [38] for dimension reduction, which not only provides a piecewise linear approximation of the data manifold but also makes the low dimensional representations still have strong discriminations. The affine subspace layer in our SAC-VLADNet can be written as:

(12) $$R_{k}=U_{k}\left(f_{ij}-c_{k}\right)=\left(U_{k}f_{ij}+\mu_{k}\right)$$

where $\mu_{k}=-U_{k}c_{k}\in R^{P\times 1}$, $U_{k}\in R^{P\times D}$ (k=1,2,⋯,K) represents the projective matrix of a specific subspace [38]. *P* represents the subspace dimension. In our SAC-VLADNet, $U_{k}$ and $\mu_{k}$ are obtained through training, instead of being directly obtained by the pre-computed $U_{k}$. $U_{k}f_{ij}+\mu_{k}$ in Equation (12) can be considered as a 1×1 convolutional layer which has the weight $\left\{U_{k}\right\}$ and the bias $\left\{\mu_{k}\right\}$, thus the conventional CNN training method can efficiently train the end-to-end affine subspace layer. The first order statistical information is written as:

(13) $$\xi_{1}\left(F_{i}\right)=\left(\begin{array}{c} \sum_{j=1}^{M}\lambda_{ij}\left(1\right)\left(U_{1}f_{ij}+\mu_{1}\right)\\ \sum_{j=1}^{M}\lambda_{ij}\left(2\right)\left(U_{2}f_{ij}+\mu_{2}\right)\\ \vdots \\ \sum_{j=1}^{M}\lambda_{ij}\left(K\right)\left(U_{K}f_{ij}+\mu_{K}\right) \end{array}\right)$$

### 3.3. The Covariance Layer

From Equation (5), it is clear to see that the original NetVLAD model only uses the first-order statistical information. The NetVLAD layer and the traditional pooling methods achieve the aggregated features from the spatial scale without considering the feature interaction between each channel. The proposed SAC-VLADNet exploits the covariance matrix to get the interactive feature which can efficiently enhance the representation ability. The final aggregated feature in the proposed network is the concatenation of the first-order and the covariance statistical information. The covariance statistical information of Equation (13) is written as:

(14) $$\left\{\begin{array}{c} \xi_{2}\left(F_{i}\right)=\sum_{k=1}^{K}\sum_{j=1}^{M}\left[\lambda_{ij}\left(k\right)\left(U_{k}f_{ij}+\mu_{k}\right)\right]{\left[\lambda_{ij}\left(k\right)\left(U_{k}f_{ij}+\mu_{k}\right)\right]}^{T}\\ \xi_{2}\left(F_{i}\right)=vec\left(\xi_{2}\left(F_{i}\right)\right) \end{array}\right.$$

where vec is the vector operation which transforms the matrix to the corresponding column vector. Based on Equation (14), we use the covariance matrix of the first order feature coding to get the interactive representation between the feature channel. Since Equation (14) is also differentiable, the covariance statistic layer can be learned by an end-to-end method.

### 3.4. The Complete SAC-VLADNet

Based on the back propagation model of the SAC-VLADNet, the proposed network can be trained by an end-to-end manner. The back propagation models of the affine subspace layer in Equation (12) and the covariance statistic layer in Equation (14) can be easily obtained. The SASAC layer is a new structure layer, and we give in detail the back propagation function of the SASAC layer in Appendix A.

For the *i*th convolutional feature $F_{i}$, the final form of SAC-VLAD coding ($\xi \left(F_{i}\right)\in R^{P(K+P)\times 1}$) is a P(K+P)-dimensional vector and written as:

(15) $$\left\{\begin{array}{c} \xi_{1}\left(F_{i}\right)=L2norm\left(\xi_{1}\left(F_{i}\right)\right)\\ \xi_{2}\left(F_{i}\right)=L2norm\left(\xi_{2}\left(F_{i}\right)\right)\\ \xi \left(F_{i}\right)=\left(\begin{array}{c} \xi_{1}\left(F_{i}\right)\\ \xi_{2}\left(F_{i}\right) \end{array}\right)\\ \xi \left(F_{i}\right)=L2norm\left(\xi \left(F_{i}\right)\right) \end{array}\right.$$

where L2norm is the L2 normalization method of a vector. From Equation (15), we could find that the final feature representation $\xi (F_{i})$ can capture both spatial aggregation information and interactive information between feature channels. This design can efficiently improve the final representation ability. Based on the derived back propagation functions, we can extend the SAC-VLAD in Equation (15) to an end-to-end deep network (SAC-VLADNet). $a_{k}$, $b_{k}$, $v_{k}$, $U_{k}$ and $\mu_{k}$ (k=1,2,⋯,K) are the learnable weights in SAC-VLADNet, and these parameters are learned by the back propagation algorithm. In the proposed SAC-VLADNet, the feed-forward procedure first computes the final softmax classification loss. Next, we compute the gradients of all the parameters and use the back propagation algorithm to update each layer in SAC-VLADNet. We use the blue and the red arrows in Figure 3 to represent the end-to-end training procedure of the SAC-VLADNet.

### 3.5. The Proposed M-SAC-VLADNet

Since the current feature coding networks (end-to-end feature coding networks [36] and non end-to-end feature coding networks [39,42]) only use the last convolutional features to compute the feature coding, these single path feature coding networks can not take full advantage of convolutional features for image classification.

Based on our newly-designed SAC-VLADNet, we further propose a novel M-SAC-VLADNet which aggregates multiple SAC-VLADNet layers for visual classification.

The M-SAC-VLADNet extracts *L* features from *L* convolutional layers. *L* features are defined as $F_{i}^{\left(1\right)},F_{i}^{\left(2\right)},\cdots ,F_{i}^{\left(L\right)}$, and $\xi \left(F_{i}^{\left(1\right)}\right),\xi \left(F_{i}^{\left(2\right)}\right),\cdots ,\xi \left(F_{i}^{\left(L\right)}\right)$ are the corresponding SAC-VLAD representations.

The final classification loss of the M-SAC-VLADNet is the standard softmax loss written as:

(16) $$loss=-\frac{1}{N}\sum_{i=1}^{N}\sum_{c=1}^{C}H\left(y_{i},c\right)\log\frac{e^{\rho_{ic}}}{\sum_{m=1}^{C}e^{\rho_{im}}}$$

where *C* is the number of categories, H{x,y}=1 is an indicator function which satisfies H{x,y}=1 if x=y, otherwise H{x,y}=0. $y_{i}$ represents the label of the *i*th image. $\rho_{ic}$ is the total prediction score:

(17) $$\rho_{ic}=\sum_{l=1}^{L}\left\{{\left(g_{c}^{\left(l\right)}\right)}^{T}\xi \left(F_{i}^{\left(l\right)}\right)+b_{c}^{\left(l\right)}\right\}$$

where ${\left[g_{1}^{\left(l\right)},g_{2}^{\left(l\right)},\cdots ,g_{C}^{\left(l\right)}\right]}^{T}$ and ${\left[b_{1}^{\left(l\right)},b_{2}^{\left(l\right)},\cdots ,b_{C}^{\left(l\right)}\right]}^{T}$ are the weight and bias of the *l*th (l=1,2,⋯,L) fully-connected (FC) layer. Equation (17) can be further written as:

(18) $$\rho_{ic}={\left(G_{c}\right)}^{T}\left[\xi \left(F_{i}^{\left(1\right)}\right);\xi \left(F_{i}^{\left(2\right)}\right);\cdots ;\xi \left(F_{i}^{\left(L\right)}\right)\right]+{\left(B_{c}\right)}^{T}$$

where $G_{c}=\left[g_{c}^{\left(1\right)};g_{c}^{\left(2\right)};\cdots ;g_{c}^{\left(L\right)}\right]$, $B_{c}=\sum_{l=1}^{L}b_{c}^{\left(l\right)}$. $G={\left[G_{1},G_{2},\cdots ,G_{C}\right]}^{T}$ and $B={\left[B_{1},B_{2},\cdots ,B_{C}\right]}^{T}$ are the weight and bias of the final softmax classifier.

Compared with the NetVLAD [36] model, which only uses the single level feature coding to train the final classifier, the proposed M-SAC-VLADNet exploits multiple SAC-VLAD codings for image classification, thus the proposed multi-path feature coding network is expected to be more discriminative.

The M-SAC-VLADNet is also an end-to-end feature coding model. We first obtain the initialization parameters in each SAC-VLADNet layer, and then train the entire M-SAC-VLADNet by an end-to-end method. Based on the back propagation algorithm, the gradient information of the softmax classifier can be used to update the parameters in each SAC-VLADNet layer. Because of this, the proposed M-SAC-VLADNet can be trained in a supervised way. We define the feed operation of the M-SAC-VLADNet as the blue arrow in Figure 4 and define the back operation of the M-SAC-VLADNet as the red arrow in Figure 4.

## 4. Experimental Results

In this section, the classification performances of the proposed SAC-VLADNet and M-SAC-VLADNet are evaluated on several image benchmarks. For a fair comparison, the parameters in NetVLAD and SAC-VLADNet are set to the same values. For other compared classification methods, we tune the corresponding parameters to get the best results. The experimental image databases include MIT [43] indoor scene database, Stanford cars [44] dataset, Caltech-UCSD Birds 200 (CUB200) [45] database and Caltech256 [46]) object database. The basic specifications of all the datasets are shown in Table 1. First, the experimental setting of the proposed network iss given. Next, we evaluate some important factors that significantly affect the image recognition rates of the proposed SAC-VLADNet. Finally, we will give some detailed experimental results of our deep network and other state-of-the-art classification models to demonstrate the superiorities of SAC-VLADNet and M-SAC-VLADNet.

### 4.1. Experimental Setting

In our experiments, we used the VGG-VD [47] network to extract the single level feature for SAC-VLADNet and the multiple levels features for M-SAC-VLADNet, All the images were resized to 448×448 pixels. We used random crop technology and random mirror technology to augment all the training images. We used the flexible and efficient deep learning library Mxnet [48] to extract the deep CNN features and implement the SAC-VLADNet and the M-SAC-VLADNet. To minimize the classification loss, the stochastic gradient descent (SGD) optimization algorithm was used.

For the proposed SAC-VLADNet, we used the VGG-VD [47] network which is pre-trained from the large scale ImageNet [40] dataset to initialize the frontal deep CNN. Then, we used the last convolutional features to learn the initialized dictionary ${\left\{c_{k}\right\}}_{k=1}^{K}$. We used the K-means algorithm in VLFeat library [49] to train the initialized dictionary. Besides, we used the affine subspace model in [38] to initialize the affine subspace parameters $U_{k}$ (k=1,2,⋯,K). We used the corresponding analytical relationships in Section 3 to initialize $a_{k}$, $b_{k}$, $v_{k}$ and $\mu_{k}$. Based on Equation (15), we obtained the final SAC-VLAD representations. Finallu, based on the obtained SAC-VLAD representations, we achieved the initial weight and bias of the last fully-connected layer by training a softmax classifier. The non end-to-end SAC-VLAD can be viewed as the initial value of the end-to-end SAC-VLADNet. Based on the back propagation algorithm, the SAC-VLADNet model can achieve the final parameters for visual classification.

For the proposed M-SAC-VLADNet, we first extracted the convolutional features of L=4 layers (Relu5_1, Relu5_2, Relu5_3 and *Pool5*) on VGG-VD [47] network to obtain four initialized SAC-VLADNet layers, and then concatenated the four SAC-VLAD representations together. Finally, based on the concatenate SAC-VLAD representations, we obtained the initial values of *G* and *B* in Figure 4 by training a softmax classifier. Based on the above initialization parameters, the M-SAC-VLADNet obtained the optimal parameters for visual classification by an end-to-end manner.

### 4.2. Analyses of Some Important Factors

In this subsection, we evaluate some important factors that affect the image recognition rate of the proposed SAC-VLADNet. When we evaluate a specific factor, we set all other factors to fixed values. We evaluate all the factors on Caltech256 [46] dataset. The experimental configuration of the Caltech256 database can be found in Section 4.6.4.

**Figure 5:**
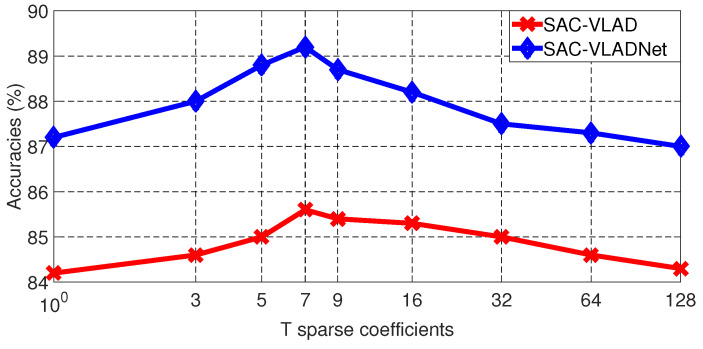
The Caltech256 classification results of the SAC-VLAD and the SAC-VLADNet with different *T*.

From Equation (10), it is clear to see that the SASAC layer only considers the largest *T* probabilities and enforces other small probabilities to be zeros. *T* is a very important factor which will affect the image recognition rates of the SAC-VLAD and the SAC-VLADNet. We compare the image recognition rates in Caltech256 [46] dataset with different *T*, we set the dictionary size (*K*) and the subspace dimension (*P*) as 128 and 128, respectively. The Caltech256 [46] image recognition rates of the SAC-VLAD and the SAC-VLADNet with different *T* are shown in Figure 5.

As shown in Figure 5, it is obvious to see that *T* should be a suitable value. If *T* is too small, such as T=1, some contributing probabilities are disregarded, which will decrease the discrimination of the SASAC layer. If *T* is too big, such as T≥32, the unreliable probabilities will also reduce the discrimination of the SASAC layer. In this experimental result, when T=7, the SAC-VLAD and the SAC-VLADNet get the best image classification performances. We can select optimal *T* for other datasets in a similar way. In a specific database experiment, we give the optimal *T*. For our M-SAC-VLADNet, *T* is set to the same value in each SAC-VLADNet layer .

**Figure 6:**
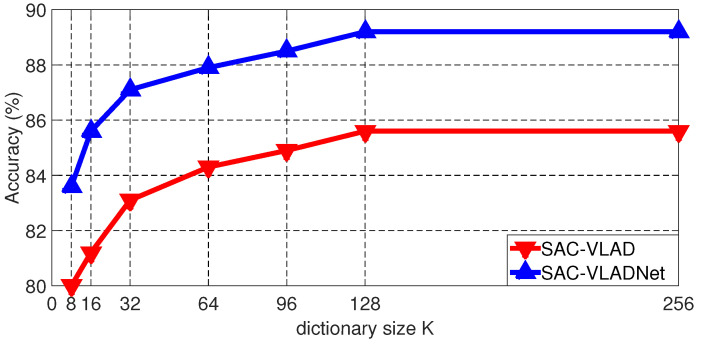
The Caltech256 classification results of the SAC-VLAD and the SAC-VLADNet with different *K*.

Dictionary size (*K*) is another pivotal parameter. The Caltech256 [46] image recognition rates of the SAC-VLAD and the SAC-VLADNet with different *K* are shown in Figure 6. We set *T* and the subspace dimension (*P*) as 7 and 128, respectively.

As shown in Figure 6, it is clear to see that, when the dictionary becomes larger, the accuracy also increases. However, after *K* is greater than a certain value, the accuracy cannot be further improved. In this experimental result, the accuracy of the SAC-VLADNet does not show apparent improvement when *K* is larger than 128. In other databases experiments, the SAC-VLADNet also gets good enough performances when K=128. In the following experiments, we set *K* as 128. For the NetVLAD model, we also set the dictionary size as 128 in the following experiments.

**Figure 7:**
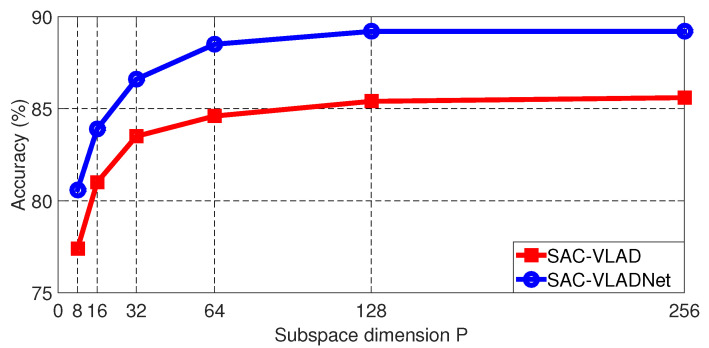
The Caltech256 classification results of the SAC-VLAD and the SAC-VLADNet with different *P*.

The final length of the SAC-VLADNet coding is determined by the subspace dimension (*P*). The Caltech256 [46] image recognition rates of the SAC-VLAD and the SAC-VLADNet with different *P* are shown in Figure 7, we set *T* and the dictionary size (*K*) as 7 and 128, respectively.

As shown in Figure 7, it is clear to see that the SAC-VLADNet does not have a good enough result when P=128. For other databases experiments, SAC-VLADNet also achieves good enough results when P=128. To make the SAC-VLADNet representation have relatively low length, we set P=128 for the following experiments. The length of the SAC-VLADNet representation is P(K+P)=128×(128+128)=32768 in the following experiments.

**Figure 8:**
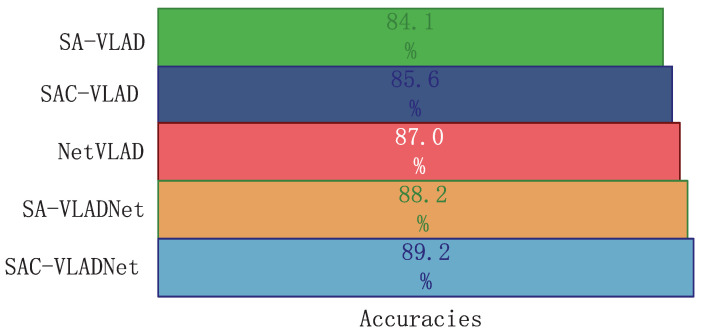
The Caltech256 classification results of the NetVLAD, the proposed models and other variants.

The SASAC layer and covariance statistic layer are two vital layers in the proposed SAC-VLADNet. To demonstrate the effects of the SASAC layer and the covariance statistic layer, we give some experimental comparisons of the variants of SAC-VLADNets. In this section, a SAC-VLADNet model that doe not have the covariance statistic layer is described as the SA-VLADNet, and the corresponding non end-to-end model is described as SA-VLAD. The image recognition rates of the NetVLAD, the proposed models and other variants in Caltech256 [46] database are shown in Figure 8. As Figure 8 shows, SAC-VLAD improves 1.5% over SA-VLAD, and SAC-VLADNet improves 1.0% over SA-VLADNet, which demonstrates the effect of the covariance statistic layer. SA-VLADNet achieves 1.2% improvement over NetVLAD, which demonstrates that the SASAC layer is also an important layer for improving discrimination. Since SASAC layer and covariance statistic layer can significantly improve the image recognition rate of the proposed SAC-VLADNet, these two layers are necessary components of the proposed deep network.

### 4.3. Statistical Test of SAC-VLADNet and NetVLAD

**Figure 9:**
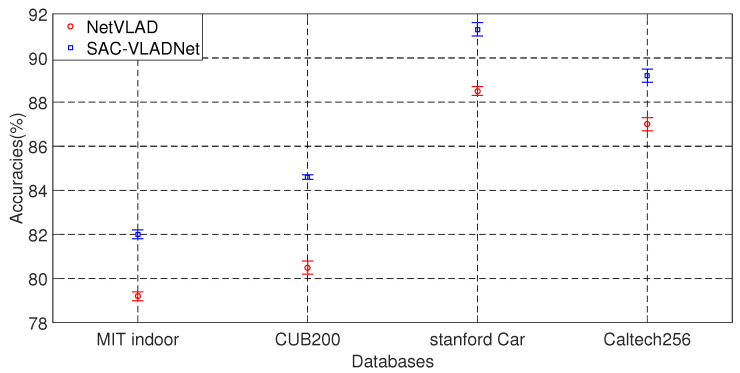
The error bars of SAC-VLADNet and NetVLAD on four databases.

In this subsection, we give the statistical test of SAC-VLADNet and NetVLAD. Figure 9 shows the error bars of SAC-VLADNet and NetVLAD on 10 different data duplicates. The error bar shows that the proposed SAC-VLADNet increases the recognition rate by 2–4% over the NetVLAD, which demonstrates the great superiority of the SAC-VLADNet. We use the Matlab t-tests function to do the statistical test of the SAC-VLADNet and the NetVLAD. The statistical test results demonstrate that the differences between the proposed SAC-VLADNet and the NetVLAD are statistically significant when significance level α=0.05.

### 4.4. Analysis of Coding Results

**Figure 10:**
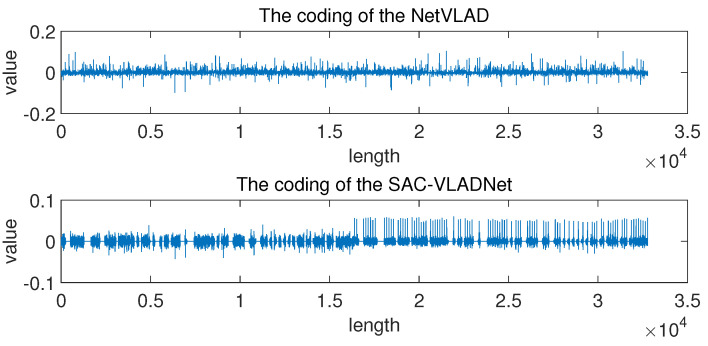
The coding results of SAC-VLADNet and NetVLAD.

In this subsection, we give some extended discussions of the coding results. We randomly select one test sample from the Caltech256 database to get the NetVLAD coding and the SAC-VLADNet coding. The coding results of SAC-VLADNet and NetVLAD are shown in Figure 10. As Figure 10 shows, the NetVLAD coding is relatively irregular, yet the SAC-VLADNet coding has some certain rules. The first half of the SAC-VLADNet coding is the first order sparse coding, and the second half of the SAC-VLADNet coding is the covariance sparse coding. The sparse and second order representations make the SAC-VLADNet coding more discriminative than the NetVLA coding. Besides, the regular SAC-VLADNet coding is better distinguished than the irregular NetVLAD coding, which enhances the representation ability of the SAC-VLADNet coding.

### 4.5. Analysis of Multi-Path Features

**Figure 11:**
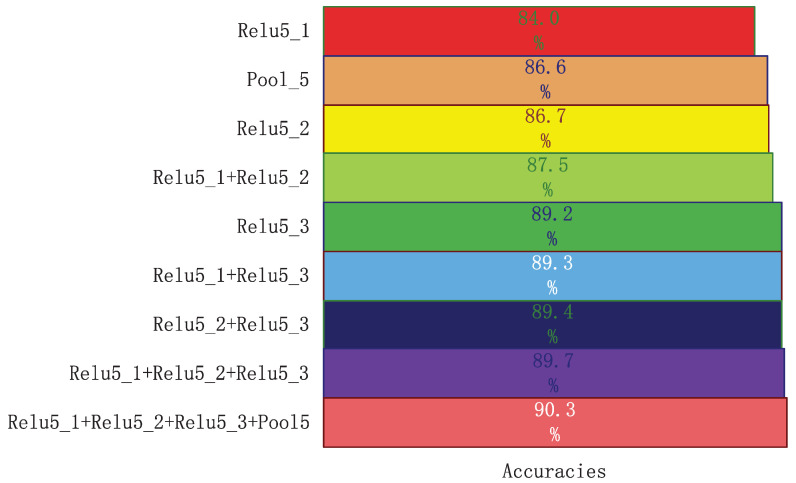
The Caltech256 classification results of four SAC-VLADNets and five M-SAC-VLADNets.

In this subsection, we analyse the effect of the M-SAC-VLADNet. M-SAC-VLADNet aggregates four SAC-VLAD representations from four convolutional layers (Relu5_1, Relu5_2, Relu5_3 and *pool5*) in VGG-VD [47] network. Figure 11 illustrates the classification performances of 4 SAC-VLADNets and 5 M-SAC-VLADNets.

In Figure 11, Relu5_1, Relu5_2, Pool5 and Relu5_3 represent four SAC-VLADNets which extract the convolutional features from Relu5_1 layer, Relu5_2 layer, Pool5 layer and Relu5_3 layer, respectively. Relu5_1+Relu5_2, Relu5_1+Relu5_3, Relu5_2+Relu5_3, Relu5_1+Relu5_2+Relu5_3 and Relu5_1+Relu5_2+Relu5_3+Pool5 are 5 M-SAC-VLADNets which extract the corresponding features from multiple convolutional layers.

As shown in Figure 11, Relu5_1+Relu5_2 is better than Relu5_1, Relu5_2 and Pool5. Besides, Relu5_1+Relu5_3, Relu5_2+Relu5_3, Relu5_1+Relu5_2+Relu5_3 and Relu5_1+Relu5_2+Relu5_3+Pool5 are better than Relu5_3. The experimental results in Figure 11 demonstrate the effects of the multi-path feature coding networks. Relu5_1+Relu5_2+Relu5_3+Pool5 utilizes four path features and is more discriminative than other M-SAC-VLADNets (Relu5_1+Relu5_2, Relu5_1+Relu5_3, Relu5_2+Relu5_3 and Relu5_1+Relu5_2+Relu5_3), which demonstrates that Relu5_1+Relu5_2+Relu5_3+Pool5 can take full advantage of multiple levels features and greatly improve the image recognition rate. In the following experiments, M-SAC-VLADNet represents Relu5_1+Relu5_2+Relu5_3+Pool5, and SAC-VLADNet represents Relu5_3.

### 4.6. Comparisons with Other State-of-the-Art Classification Models

In this subsection, the experimental results of the proposed model and other state-of-the-art models on each dataset will be given.

#### 4.6.1. MIT Indoor Recognition

MIT [43] indoor scene database is a challenging indoor scene dataset. This dataset consists of 15,620 indoor scene samples of 67 classes. The common training/test division in [43] is used to obtain the scene recognition results.

In the MIT [43] indoor scene dataset, the optimal *T* is 7, the compared models in this dataset include FV-CNN [39], FC-CNN [39], Bilinear CNN (B-CNN) [50], Task driven pooling (TDP) [51], CaffeNet [1], directed acyclic graph CNN (DAG-CNN) [52], Caffe-DAG [52] and NetVLAD [36]. The original FV-CNN [39] coding model uses the multi-scale input images to obtain the FV representations. However, the proposed SAC-VLADNet uses the single-scale images with 448×448 pixels to get the SAC-VLAD representations. To get a fair result, in our comparative experiment, FV-CNN model utilizes the single-scale images with 448×448 pixels to get the FV representation. Table 2 shows the image recognition rates of the proposed model and other methods on MIT-indoor [43] database. As Table 2 shows, since VGG-VD [47] network can extract deeper CNN features than the AlexNet [1], VGG-VD [47] based methods are much better than AlexNet [1] based methods. Compared with FC-CNN [39], FV-CNN [39], TDP [51] and DAG-CNN [52], which are the VGG-VD methods, our SAC-VLADNet has obvious advantages. Besides, the SAC-VLADNet improves 2.8% over the NetVLAD [36] and 2.4% over the B-CNN which are end-to-end trained deep networks, this classification result shows the effectiveness of the proposed deep feature coding network. M-SAC-VLADNet achieves 0.9% improvement over SAC-VLADNet and has obvious advantages over other CNN methods, thus the proposed M-SAC-VLADNet is very effective for scene classification.

#### 4.6.2. CUB200 Classification

Caltech-UCSD Birds 200 (CUB200) [45] is a widely used bird image database. CUB200 dataset consists of 11,788 bird images from 200 bird categories, and the training and test sets in this database are roughly equal. Besides, this dataset has detail part annotation and bounding box annotation. Bird images always have different poses and viewpoints, and the background will affect the estimation of the birds, thus classifying bird categories is very challenging.

In the CUB200 database, the optimal *T* is 5. The compared models include fisher vector (FV) coding [34], part R-CNN [53], part stacked CNN (PS-CNN [54]), deep LAC [55], FV-CNN [39], Probabilistic Collaborative Representation classification (ProCRC) [56], NetVLAD [36], Neural Activation Constellations (NAC) [57], without part annotations (WPA) [58], Multiple Granularity Descriptors (Multi-grained) [59], Random Maclaurin compact bilinear pooling (CBP-RM) [60], Tensor sketch compact bilinear pooling (CBP-TS) [60], B-CNN [50], low rank bilinear pooling (LRBP) [61] and semantic part detection and abstraction CNN (SPDA-CNN) [62].

The CUB200 database also gives the annotations of Part and bounding box (bbox), yet our methods only utilize the class information and not consider annotation of part and bounding box.

As Table 3 shows, the traditional FV [34] coding method uses the SIFT feature to compute the FV coding, thus the traditional FV [34] coding method is significantly worse than other CNN methods. Part R-CNN [53], PS-CNN [54] and Deep LAC [55] are based on AlexNet [1], and these AlexNet methods are usually worse than other VGG-VD [35] methods. Considering the VGG-VD [47] methods, our end-to-end SAC-VLADNet achieves 7.6% improvement over our non end-to-end SAC-VLAD, which shows the great superiority of the end-to-end training manner in the proposed network. Besides, our SAC-VLADNet is obviously better than FV-CNN [39], ProCRC [56], NAC [57], Multi-grained [59] and WPA [58]. Compared with the NetVLAD [36], our SAC-VLADNet achieves 4.1% improvement, which shows the effects of the new structure end-to-end layers. B-CNN, CBP-RM [60], CBP-TS [60] and LRBP [61] are state-of-the-art end-to-end models on CUB200 database, and our end-to-end SAC-VLADNet is comparable to these end-to-end methods. Based on the VGG-VD [47] network, SPDA-CNN [62] learns a better part detectors and achieves 84.6% recognition rate. Compared with the SPDA-CNN [62] model, our M-SAC-VLADNet achieves 0.9% improvement and doesn’t require extra annotations of part and bounding box. The highest accuracy of the M-SAC-VLADNet in Table 3 demonstrates that our multi-path feature coding network is very effective for bird classification.

#### 4.6.3. Car Categorization

Stanford [44] car database consists of 16,185 car samples of 196 classes. This dataset is split into 8144 training car images and 8041 test car images. The widely used training and test divisions in [44] are used to obtain the car categorization performances.

In the car database, the optimal *T* is 3, the compared models include FV coding [34], revisiting the fisher vector (RFV [63]), FV-CNN [39], NetVLAD [36], CBP-RM [60], CBP-TS [60], B-CNN [50], LRBP [61] and boosted CNN (BoostCNN [64]). Table 4 shows the Stanford cars recognition rates of our network and other competing models.

As Table 4 shows, the SIFT feature methods (FV coding [34] and RFV [63]) are significantly worse than the other CNN methods. Compared with the NetVLAD, our SAC-VLADNet achieves 2.8% improvement, which demonstrates that our new structure end-to-end layers can efficiently improve the image classification performance. Besides, our SAC-VLADNet is comparable to CBP-RM [60], CBP-TS [60], B-CNN [50] and LRBP [61] which are end-to-end deep models. BoostCNN [64] is a state-of-the-art CNN model on Car dataset. Our M-SAC-VLADNet achieves 0.4% improvement over BoostCNN [64] and is obviously better than the other CNN methods, which shows the advantage of the new structure M-SAC-VLADNet in car categorization.

#### 4.6.4. Caltech256 Classification

Caltech256 [46] is a massive object image database. This database consists of 256 object categories with at least 80 samples per classer. The total number of this database is 30,680. Following the widely-used experimental setting, we randomly select 60 images per class as the training set and use the remaining images as the test set. To get a fair results, we run our methods 10 times for each partition and report the average classification accuracies.

In Caltech256 database, the optimal *T* is 7. The compared models include sparse coding spatial pyramid matching (ScSPM [26]), locality constrained coding (LLC [30]), FV-CNN [39], NAC [57], FC-CNN [39], Deep Spatial Pyramid (DSP) [42], ProCRC [56] and NetVLAD. Table 5 shows the Caltech256 recognition rates of our deep network and the other competing models.

As Table 5 shows, the traditional SIFT feature coding methods (ScSPM [26] and LLC [30]) are significantly worse than the CNN feature coding methods. Compared with FV-CNN [39], NAC [57], FC-CNN [39], DSP [42] and ProCRC [56], our SAC-VLADNet gets significant improvement. Compared with the NetVLAD [36], our SAC-VLADNet achieves 2.2% improvement, which demonstrates the superiorities of the newly-designed end-to-end layers. Besides, our M-SAC-VLADNet achieves at least 1.1% improvement over the others, which demonstrates the superiority of our multi-path feature coding network in object classification.

### 4.7. Running Speed Comparison

Table 6 gives the training and test speeds (samples per second) of the SAC-VLADNet, the M-SAC-VLADNet, the VGG-VD and the NetVLAD. In the training stage, since the SAC-VLADNet uses the concatenation of the first-order and the covariance statistics, the SAC-VLADNet is more time-consuming than the NetVLAD [36] which only computes the first-order VLAD coding. Besides, since the VGG-VD [47] has multiple high-dimensional fully connected layers, SAC-VLADNet is faster than VGG-VD. Since the M-SAC-VLADNet aggregates multiple feature coding layers, our multi-path network is slower than the SAC-VLADNet, and the running speed of the M-SAC-VLADNet is similar to that of the VGG-VD. In the test stage, the proposed SAC-VLADNet is slightly slower than the NetVLAD and faster than the VGG-VD. Although the SAC-VLADNet and the M-SAC-VLADNet are slower than the NetVLAD, considering the SAC-VLADNet and the M-SAC-VLADNet have the better image classification performances, the proposed deep networks are still very effective.

## 5. Conclusions

In this work, we propose a sparsely-adaptive and covariance VLAD (SAC-VLAD) coding method which is more discriminative than the original VLAD coding method. Based on the back propagation models, the SAC-VLAD coding method is extended to an end-to-end SAC-VLADNet. We further propose an end-to-end multi-path SAC-VLADNet (M-SAC-VLADNet) which aggregates multiple SAC-VLADNet layers for visual classification. Our models can efficiently embed the domain knowledge of the feature coding into the deep convolutional neural network. The experimental comparisons demonstrate that the our model is very competitive for visual classification.

## Figures and Tables

**Table 1:** The basic specifications of all the databases.

	Sample Number	Classes	*T*	*K*	*P*
MIT indoor	15620	67	7	128	128
CUB200	11788	200	5		
Standford Car	16185	196	3		
Caltech256	30680	256	7		

**Table 2:** The accuracies (%) on the MIT-indoor dataset.

Methods	Features	Accuracies (%)
CaffeNet [1]	AlexNet	59.5
Caffe-DAG [52]	AlexNet	64.6
FC-CNN [39]	VGG-VD	68.1
FV-CNN [39]	VGG-VD	76.0
TDP [51]	VGG-VD	75.6
DAG-CNN [52]	VGG-VD	77.5
NetVLAD [36]	VGG-VD	79.2
B-CNN [50]	VGG-VD	79.6
SAC-VLAD	VGG-VD	78.6
SAC-VLADNet	VGG-VD	82.0
M-SAC-VLADNet	VGG-VD	82.9

**Table 3:** The accuracies (%) on the CUB200 dataset.

Methods	Features	Train	Test	Acc (%)
FV coding [34]	SIFT	n/a	n/a	18.8
Part R-CNN [53]	AlexNet	Box+Part	n/a	73.9
PS-CNN [54]	AlexNet	Box+Part	Box	76.6
Deep LAC [55]	AlexNet	Box+Part	Box	80.2
FV-CNN [39]	VGG-VD	n/a	n/a	71.3
ProCRC [56]	VGG-VD	n/a	n/a	78.3
NetVLAD [36]	VGG-VD	n/a	n/a	80.5
NAC [57]	VGG-VD	n/a	n/a	81.0
Multi-grained [59]	VGG-VD	n/a	n/a	81.7
WPA [58]	VGG-VD	Box	n/a	82.0
CBP-RM [60]	VGG-VD	n/a	n/a	83.9
B-CNN [50]	VGG-VD	n/a	n/a	84.0
CBP-TS [60]	VGG-VD	n/a	n/a	84.0
LRBP [61]	VGG-VD	n/a	n/a	84.2
SPDA-CNN [62]	VGG-VD	Box+Part	Box	84.6
SAC-VLAD	VGG-VD	n/a	n/a	77.0
SAC-VLADNet	VGG-VD	n/a	n/a	84.6
M-SAC-VLADNet	VGG-VD	n/a	n/a	85.5

**Table 4:** The accuracies (%) on the Stanford cars dataset.

Methods	Features	Accuracies (%)
FV coding [34]	SIFT	59.2
RFV [63]	SIFT	82.7
FV-CNN [39]	VGG-VD	85.7
NetVLAD [36]	VGG-VD	88.5
CBP-RM [60]	VGG-VD	89.5
CBP-TS [60]	VGG-VD	90.2
B-CNN [50]	VGG-VD	90.6
LRBP [61]	VGG-VD	90.9
BoostCNN [64]	VGG-VD	92.1
SAC-VLAD	VGG-VD	84.1
SAC-VLADNet	VGG-VD	91.3
M-SAC-VLADNet	VGG-VD	92.5

**Table 5:** The accuracies (%) on the Caltech256 dataset.

Methods	Features	Accuracies (%)
ScSPM [26]	SIFT	40.1
LLC [30]	SIFT	47.7
FV-CNN [39]	VGG-VD	81.2
NAC [57]	VGG-VD	84.1
FC-CNN [39]	VGG-VD	85.1
DSP [42]	VGG-VD	85.5
ProCRC [56]	VGG-VD	86.1
NetVLAD [36]	VGG-VD	87.0
SAC-VLAD	VGG-VD	85.6
SAC-VLADNet	VGG-VD	89.2
M-SAC-VLADNet	VGG-VD	90.3

**Table 6:** The running speed (samples/second) of the related methods.

	Train	Test
VGG-VD	13.95	103.8
NetVLAD	24.7	114.8
SAC-VLADNet	22.0	105.3
M-SAC-VLADNet	14.3	98.8

## Appendix A. The Back Propagation Function of SASAC Layer

For each *k* (k=1,2,⋯,K), Equation (10) is equivalent to the following three expressions:

(A1) $$\beta_{ij}\left(k\right)=e^{-\left|\right|a_{k}.*f_{ij}+b_{k}{\left|\right|}_{2}^{2}+v_{k}}$$

(A2) $$\gamma_{ij}\left(k\right)=\left\{\begin{array}{c} \beta_{ij}\left(k\right),\;k\in S_{T}\left(f_{ij}\right)\\ 0,\;otherwise \end{array}\right.$$

(A3) $$\lambda_{ij}\left(k\right)=\frac{\gamma_{ij}\left(k\right)}{\sum_{k^{\prime }=1}^{K}\gamma_{ij}\left(k^{\prime }\right)}$$

Equation (A2) can be considered as a variant of the max pooling layer. In max pooling layer, the largest value is held and the remaining values are ignored. In Equation (A2), the largest *T* values are held and the remaining values are set to be zeros. Equation (A3) is a normalized layer which can obtain normalized weight coefficients. In this paper, the final classification loss is defined as *J*. For each *k* (k=1,2,⋯,K), the gradient of the loss *J* with respect to the output of the SASAC layer is defined as $\frac{\partial J}{\partial \lambda_{ij}\left(k\right)}$. When $\frac{\partial J}{\partial \lambda_{ij}\left(k\right)}$ is obtained, by using the chain rule, the gradients of $\gamma_{ij}\left(k\right)$ and $\beta_{ij}\left(k\right)$ are derived as:

(A4) $$\frac{\partial J}{\partial \gamma_{ij}\left(k\right)}=\frac{\frac{\partial J}{\partial \lambda_{ij}\left(k\right)}\sum_{m\neq k}\gamma_{ij}\left(m\right)-\sum_{k^{\prime }\neq k}\frac{\partial J}{\partial \lambda_{ij}\left(k^{\prime }\right)}\gamma_{ij}\left(k^{\prime }\right)}{{\left[\sum_{m=1}^{K}\gamma_{ij}\left(m\right)\right]}^{2}}$$

(A5) $$\frac{\partial J}{\partial \beta_{ij}\left(k\right)}=\left\{\begin{array}{c} \frac{\partial J}{\partial \gamma_{ij}\left(k\right)},\;k\in S_{T}\left(f_{ij}\right)\\ 0,\;otherwise \end{array}\right.$$

Based on $\beta_{ij}\left(k\right)$ (k=1,2,⋯,K), the gradients of the loss with respect to the layer input and trainable parameters ($a_{k}$, $b_{k}$ and $c_{k}$) can be obtained. The following contents will present these back propagation functions.

Gradient of $f_{ij}$: The gradient of *J* with respect to $f_{ij}$ can be obtained by:

(A6) $$\frac{\partial J}{\partial f_{ij}}=\sum_{k=1}^{K}\frac{\partial J}{\partial \beta_{ij}\left(k\right)}\frac{\partial \beta_{ij}\left(k\right)}{\partial f_{ij}}$$

Based on Equation (A1), $\frac{\partial \beta_{ij}\left(k\right)}{\partial f_{ij}}$ is derived as:

(A7) $$\frac{\partial \beta_{ij}\left(k\right)}{\partial f_{ij}}=-2a_{k}.*\left(a_{k}.*f_{ij}+b_{k}\right)\beta_{ij}\left(k\right)$$

Based on Equations (A6) and (A7), the gradient of $f_{ij}$ is derived as:

(A8) $$\frac{\partial J}{\partial f_{ij}}=\sum_{k=1}^{K}-2\frac{\partial J}{\partial \beta_{ij}\left(k\right)}a_{k}.*\left(a_{k}.*f_{ij}+b_{k}\right)\beta_{ij}\left(k\right)$$

Gradients of $a_{k}$, $b_{k}$ and $v_{k}$: The gradients of *J* with respect to $a_{k}$, $b_{k}$ and $v_{k}$ can be obtained by:

(A9) $$\left\{\begin{array}{c} \frac{\partial J}{\partial a_{k}}=\sum_{j=1}^{M}\frac{\partial J}{\partial \beta_{ij}\left(k\right)}\frac{\partial \beta_{ij}\left(k\right)}{\partial a_{k}}\\ \frac{\partial J}{\partial b_{k}}=\sum_{j=1}^{M}\frac{\partial J}{\partial \beta_{ij}\left(k\right)}\frac{\partial \beta_{ij}\left(k\right)}{\partial b_{k}}\\ \frac{\partial J}{\partial v_{k}}=\sum_{j=1}^{M}\frac{\partial J}{\partial \beta_{ij}\left(k\right)}\frac{\partial \beta_{ij}\left(k\right)}{\partial v_{k}} \end{array}\right.$$

Based on Equation (A1), $\frac{\partial J}{\partial a_{k}}$, $\frac{\partial J}{\partial b_{k}}$ and $\frac{\partial J}{\partial v_{k}}$ are derived as:

(A10) $$\left\{\begin{array}{c} \frac{\partial \beta_{ij}\left(k\right)}{\partial a_{k}}=-2f_{ij}.*\left(a_{k}.*f_{ij}+b_{k}\right)\beta_{ij}\left(k\right)\\ \frac{\partial \beta_{ij}\left(k\right)}{\partial b_{k}}=-2\left(a_{k}.*f_{ij}+b_{k}\right)\beta_{ij}\left(k\right)\\ \frac{\partial \beta_{ij}\left(k\right)}{\partial v_{k}}=\beta_{ij}\left(k\right) \end{array}\right.$$

Based on Equations (A9) and (A10), the gradients of $a_{k}$, $b_{k}$ and $v_{k}$ are derived as:

(A11) $$\left\{\begin{array}{c} \frac{\partial J}{\partial a_{k}}=\sum_{j=1}^{M}-2\frac{\partial J}{\partial \beta_{ij}\left(k\right)}f_{ij}.*\left(a_{k}.*f_{ij}+b_{k}\right)\beta_{ij}\left(k\right)\\ \frac{\partial J}{\partial b_{k}}=\sum_{j=1}^{M}-2\frac{\partial J}{\partial \beta_{ij}\left(k\right)}\left(a_{k}.*f_{ij}+b_{k}\right)\beta_{ij}\left(k\right)\\ \frac{\partial J}{\partial v_{k}}=\sum_{j=1}^{M}\frac{\partial J}{\partial \beta_{ij}\left(k\right)}\beta_{ij}\left(k\right) \end{array}\right.$$
